# A Mobile App (AMOR Mama) for Women With Breast Cancer Undergoing Radiation Therapy: Functionality and Usability Study

**DOI:** 10.2196/24865

**Published:** 2021-10-13

**Authors:** Flávia Oliveira de Almeida Marques da Cruz, Edison Tostes Faria, Pabblo Cardelino Ghobad, Leandro Yukio Mano Alves, Paula Elaine Diniz dos Reis

**Affiliations:** 1 Interdisciplinary Laboratory of Research Applied to Clinical Practice in Oncology School of Health Sciences University of Brasilia (UnB) Brasilia Brazil; 2 Faculty of Medicine University of Brasilia (UnB) Brasilia Brazil; 3 Federal Senate of Brazil Brasilia Brazil; 4 State University of Rio de Janeiro (UERJ) Rio de Janeiro Brazil; 5 Department of Nursing School of Health Sciences University of Brasilia (UnB) Brasilia Brazil

**Keywords:** mobile applications, health education, nursing care, oncology nursing, educational technology, breast neoplasms, radiation therapy

## Abstract

**Background:**

Mobile apps targeting women with breast cancer can facilitate access to information, improve well-being, and record reports of treatment-related symptoms. However, it is important to confirm the benefits of these apps before they are used as a tool in clinical care.

**Objective:**

The aim of this study was to evaluate the functionality and the usability of a mobile app created to guide and monitor patients with breast cancer undergoing radiation therapy.

**Methods:**

The evaluation process of the mobile app was performed in 2 steps with 8 professionals, including nurses, physician, medical physicists, and communication networks engineer. The first step was the focus group, which allowed obtaining suggestions proposed by the participants regarding the improvement of the mobile app. The second step was the individual filling in of an evaluation tool to obtain objective measures about the mobile app. A minimum concordance index of 80% was considered to ensure the adequacy of the material.

**Results:**

After the mobile app was evaluated by 8 professionals, only 1 item of the evaluation tool, that is, concerning the potentiality of the app to be used by users of different educational levels, obtained a concordance index <80%.

**Conclusions:**

The mobile app titled “AMOR Mama” was considered suitable, which suggests its contribution to an educational health technology to guide and monitor patients with breast cancer undergoing radiation therapy. More studies with this target population should be carried out to assess the performance and quality of the mobile app during its use.

## Introduction

Cancer is a leading cause of disability and mortality worldwide, affecting more than 18 million people each year [1]. Breast cancer is the most frequent among women, with approximately 2.1 million new cases diagnosed in 2018 [2]. Radiation therapy is a treatment modality that uses ionizing radiation to prevent the multiplication of tumor cells and to determine their death by delivering radiation to the tumor with the least possible damage to the healthy surrounding tissues [3]. When the breast region is exposed to radiation therapy, the most common side-effects are pain, radiation dermatitis, restricted mobility, local sensory alteration, and fatigue [4]. In addition to the physical factors related to the disease and treatment, it is worth mentioning the negative psychological impacts caused by breast cancer, mainly on the perception of body image, sexuality, and femininity [5]. Therefore, nurses need to perform their care role related to cancer and therapy effects as a facilitator in the coping process when providing comprehensive and individualized care to patients.

Mobile apps can help individuals in managing their own health and well-being as well as in promoting healthy lifestyles and obtaining quick access to important information. The use of mobile technologies has brought innovative possibilities for improving health care provision [6]. A study reported the development of a mobile app prototype to assist health care professionals in the prevention and classification of pressure ulcers. The app was evaluated for technical and functional quality by experts in the field of computing and nursing, and it was considered adequate with respect to reliability, usability, efficiency, functionality, and portability [7]. Another study described the development and evaluation of an app to support decision-making in the process of early mobilization of critical patients admitted to an intensive care unit. The app was evaluated by 58 physiotherapy undergraduate students, and it was considered practical and easy to understand and manipulate [8]. A separate study focused on the development and testing of a tablet app to collect computerized data on tobacco use among psychiatric patients and the general population, thereby replacing paper surveys. This app prevented human errors, allowed automatic tabulation, and made the interviews less tiring [9]. A recent systematic review suggests that apps for women with breast cancer can facilitate access to information and improve patients’ well-being as well as improve the report of symptoms and adverse treatment-related effects. However, the real benefit of using apps is still uncertain since the apps are often introduced into clinical care before necessary research is carried out to confirm their benefits [10]. In this context, this study aimed to evaluate the functionality and the usability of a mobile app created to guide and monitor patients with breast cancer undergoing radiation therapy.

## Methods

### Study Design

This is a methodological research focusing on the evaluation of the functionality and usability of a mobile app prototype for patients with breast cancer undergoing radiation therapy. The app was entitled “AMOR Mama” and features content of an educational manual developed and validated in a previous study called as “Orientations Manual: breast radiation therapy” [11]. The manual contains 36 pages, divided into pretextual items (cover, back cover, cataloguing sheet, index, presentation, and registration card), textual items (chapters on radiation therapy, stages of treatment, adverse effects of radiation therapy, and how to prevent them), and posttextual items (latest information, weekly diary, and bibliographical references).

The features and functionality of the app were then defined and associated with the textual content of the validated manual. Among them, we can mention user registration; registration of events in calendar and medications in use, including the possibility of generating automatic reminders through notifications; calculation of the water intake target according to the user’s weight; recording and monitoring of signs and symptoms related to breast radiation therapy, as well as recommendations and necessary care. The data registration was planned to be used in the preparation of reports that can be forwarded to the health team involved in the user’s care, thus facilitating their monitoring.

We aimed to develop a prototype of an app with relevant information and useful resources to encourage interaction between the user and the app. It has 5 menus that list the main sections: Home Page, About Radiation Therapy, Diary, Schedule, and Settings. One of the features of “Settings” is the tutorial that was designed to be the user’s initial experience during their first access.

### Methodological Reference

We chose to use the standard published by the International Organization of Standardization and the International Electrotechnical Commission, revised and translated by the Brazilian Association of Technical Standards, named as NBR ISO/IEC 9126-1. This standard describes a quality model for software products, which is composed of 2 parts: internal and external quality and quality in use [12]. The internal and external quality of the software refer to a group of characteristics that the model must present to be able to meet the user’s needs, such as functionality, usability, reliability, efficiency, maintainability, and portability [12]. Quality in use involves 4 characteristics: effectiveness, productivity, safety, and satisfaction. It is related to the evaluation of the final product from the user’s point of view, which is measured according to the performance obtained by the target audience when using the software. It is necessary to guarantee the internal and external quality of software to obtain the quality in use [12].

This study carried out the internal and external quality of the software, and the characteristics of functionality and usability were investigated since what is being evaluated is a prototype of an app, that is, a set of screens that present the interface and the proposed functions for the app and not the software that is already developed. The functionality is related to the potentiality of the app to offer the functions and resources that meet the needs of users, that is, the degree to which the software is able to provide functions that satisfy such needs [12,13]. Usability is achieved when the software has intelligibility, apprehensibility, and operability, that is, when it is easy to understand, to learn, and to operate [12]. Good usability is directly related to the success of the software in which the user is able to employ the functions and resources offered by the app with ease and efficiency [13].

### Process of the Mobile App Design Assessment

**Figure 1:**
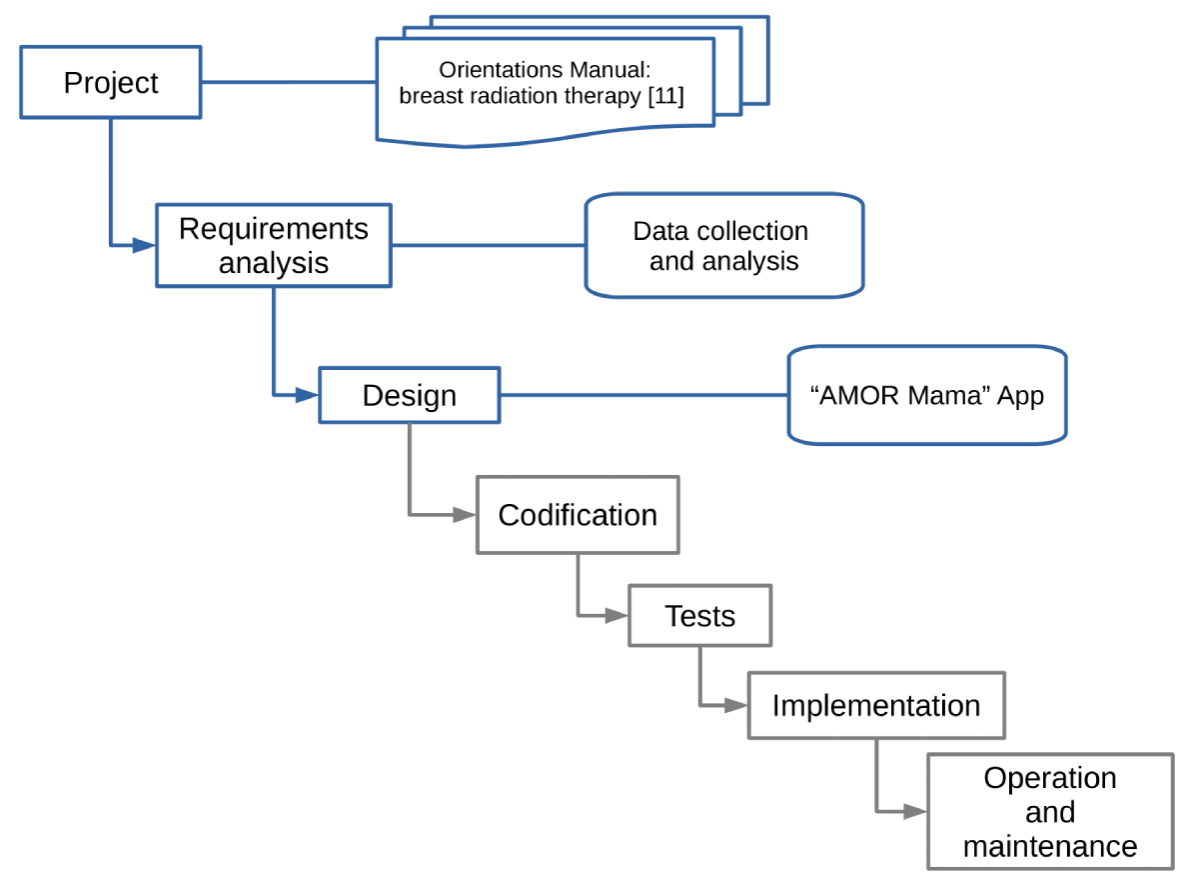
Phases of software development. The blue boxes show the steps that were developed in this study while the gray boxes show the steps that will be developed in a future study.

This study carried out actions that involve the development of software up to the modeling phase, that is, the elaboration and analysis of a project. A project is the representation of the aspects of software that are visible to the user such as the interface layout and the display formats on the screen [13]. Figure 1 shows the software development cycle: the blue boxes show the steps that were developed in this study while the gray boxes show the steps that will be developed in a future study.

The evaluation process of the app prototype was performed in 2 steps. The first one was the focus group, which allowed obtaining the suggestions proposed by the participants regarding the improvement of the app. The second step was the individual filling in of an evaluation tool to obtain objective measures about the mobile app. The researcher presented the display screens of the mobile app prototype during the meeting of the focus groups, with the aid of a multimedia projector. The interface was exhibited and its functionality demonstrated.

### Research Participants

Intentional nonprobabilistic sampling was used, considering that the sample must be defined according to the study objectives. The factors used to choose these professionals were then determined according to their academic degree, specialization, scientific production, knowledge, and time of work, according to the adaptation of the evaluation system defined in the Fehring model (1987) [14]. The recommendation is that at least 8 participants perform the design assessment of the mobile app [15]. The invitation was sent by email to 20 professionals qualified to participate in the study. Six professionals expressed interest in participating; however, they were not available during the days and times foreseen for the focus group, while 6 other professionals did not respond to the invitation even after 3 attempts to contact them. Therefore, 8 professionals participated in this research who attended the focus group at an agreed date and time.

### Data Collection and Analysis

The data were collected from November to December 2019. There were difficulties in scheduling a single meeting with all the professionals, given the specificities and individual demands. Therefore, 2 focus groups were held at different dates and times, previously agreed with the participants. The suggestions obtained through the focus groups were transcribed and analyzed by the researchers both within each focus group and comparatively between the 2 focus groups carried out to identify suggestion patterns. The quantitative data were collected using a Likert scale evaluation tool, which allowed objective measures to be obtained in relation to participants’ opinions about the app prototype. This tool was adapted from a preexisting questionnaire [16], with the necessary changes and adjustments in relation to the theme addressed in this study. It has 5 levels of judgment regarding the items: no, in parts, not sure, yes, and certainly. The options yes and certainly were grouped and represent the adequacy of the item. To determine the adequacy of the app prototype, a minimum concordance index of 80% was determined among the participants [17]. The set composed of options yes (Y) and certainly (C) should comprise at least 80% of professionals’ responses to determine the quality of the app. Thus, concordance index was calculated by the mathematical formula: (Y+C)×100/total responses. Each item that obtained concordance index below 80% was analyzed and the app was improved according to the participants’ suggestions, scientific literature, and clinical evidence.

### Ethical Aspects of the Research

This research project was sent to the Research Ethics Committee of the College of Health Sciences of the University of Brasilia (CEP/FS-UnB) and approved by Opinion 2.608.031, CAAE: 71348417.7.0000.0030.

## Results

### Profile of the Research Participants

For the mobile app evaluation, the sample consisted of 8 professionals, including nurses, doctor, medical physicists, and communication network engineer. Regarding gender, 5 were women and 3 were men. The age of the professionals ranged from 24 to 43 years (mean 32 [SD 6] years), while educational background ranged from 2 to 20 years (mean 8 [SD 6] years) and that of working in the subject area from 1 to 20 years (mean 7 [SD 6] years). Regarding the highest academic degree, 6 participants had master’s degrees and 2 were specialists.

### Process of the Mobile App Design Assessment

The professionals’ evaluations (N=8) were analyzed quantitatively. In the evaluation tool, the options yes and certainly were grouped to represent the adequacy of the item. Table 1 shows the judgment of the experts and the concordance index for each item of the evaluation tool. The focus was to investigate the participants’ opinion regarding the usability of the mobile app and the possibility of it being recommended for wide use by patients with breast cancer undergoing radiotherapy. Only item 19 did not reach the minimum concordance index established, reaching 75% of agreement among the experts. All other items reached a minimum concordance index of 80% (range 88%-100%).

In item 19, the professional who marked the option “in parts” gave the following justification for their choice: “I believe that the initial guidelines for tomography and symptoms—as presented in the text—are not simple for patients with different levels of education (E4).”

**Table 1:** Professionals’ assessment regarding the usability and functionality of the mobile app prototype.

Evaluation items	Experts’ levels of judgement (N=8)					Concordance index (%)^a^
	No	In parts	Not sure	Yes	Certainly	
1. It seems to be easy to understand the concept and manipulation of the app.	0	0	0	1	7	100
2. The app appears to be self-explanatory.	0	0	0	0	8	100
3. The tutorial is useful to optimize the use of the app.	0	0	0	1	7	100
4. The organization of the menu is logical.	0	0	0	1	7	100
5. Icons are clear and intuitive.	0	0	0	1	7	100
6. The look of the app (design and layout) is attractive.	0	0	0	2	6	100
7. It appears to be easy to navigate the app screens.	0	0	0	1	7	100
8. The sequence of actions in the app is consistent.	0	0	0	1	7	100
9. The app has useful functions.	0	0	0	2	6	100
10. The app appears to be accurate in performing its functions.	0	0	0	1	7	100
11. It seems easy to find information in the app.	0	0	0	2	6	100
12. It seems easy to insert personal information in the app.	0	0	0	2	6	100
13. It seems easy to insert events and medications into the app.	0	0	0	3	5	100
14. The texts in the app are easy to read.	0	0	0	2	6	100
15. The illustrations are important to complement the texts and facilitate the use of the app.	0	0	0	0	8	100
16. The screens are visually pleasing and seem to encourage the use of the app.	0	0	0	1	7	100
17. Using the app seems simple and accessible.	0	0	0	2	6	100
18. It seems possible to maintain interaction between the user and the app.	0	0	0	2	6	100
19. The app can be used by users of different educational levels.	0	1	1	4	2	75
20. I believe that most users would learn how to use the app.	0	0	1	2	5	88
21. The app can be used anywhere, such as at home, hospital, or street.	0	0	1	1	6	88
22. I would recommend the app to be used by women with breast cancer undergoing radiation therapy.	0	0	0	0	8	100

^a^Concordance index was calculated by the sum of yes and certainly judgments: (Yes+Certainly)×100/total of responses.

### Research Participants’ Suggestions

After analyzing the suggestions of the focus groups, the changes proposed by the participants and the researchers’ decisions regarding whether to accept such changes in the final version of the app are described in Table 2. It is worth mentioning that the communication networks engineer was the professional that suggested the most number of changes, which are mainly related to the functions and resources of the app.

**Table 2:** Summary of the participants’ proposed changes and the researchers’ decision.

Items (list of changes)		Participants’ changes proposed	Decision
Icon		Change the app title “A.M.O.R. Mama” to “AMOR Mama”	Accepted
**Tutorial**			
	1	Replace “About the app” (from Portuguese *Sobre o app*) with “Tutorial”	Accepted
	2	Add an introduction screen to the tutorial	Accepted
	3	Add information about the home page	Accepted
	4	Replace “Clicking on this icon at the navigation bar (…)” (from Portuguese *Clicando neste ícone na barra de navegação* *(...)*) with “Here you will find your diary, where you will be able to insert (…)” (from Portuguese *Aqui você encontrará o seu Diário, onde poderá registrar (...)*)	Accepted
	5	Replace “Clicking on this icon at the navigation bar (…)” with “On this icon you will access your calendar, where you will be able to insert (…)” (from Portuguese *Neste ícone você terá acesso a sua Agenda, onde poderá cadastrar (...)*)	Accepted
	6	Replace “Clicking on this icon at the navigation bar (…)” with “Here you will be able to change your personal data and obtain information about (…)” (from Portuguese *Aqui você poderá alterar seus dados pessoais e obter informações sobre (...)*)	Accepted
	7	Add a tutorial completion screen	Accepted
Home page		Add a home page that includes the information recorded daily in the schedule	Accepted
**About radiation therapy**			
	1	Add explanatory videos recorded by the team and in the real treatment environment	Accepted
	2	Add links to informational content available on the web	Not accepted
	3	In “Steps of the Treatment” (from Portuguese *Etapas do Tratamento*), include the possibility of adding the date and time of the tomography	Accepted
	4	In “Adverse Effects” (from Portuguese *Efeitos Adversos*), add a direct link to the schedule, allowing the insertion of appointments already scheduled at the institution	Accepted
**Diary**			
	1	Add “Export data” (from Portuguese *Exportar dados*) next to the download icon	Accepted
	2	Add the possibility to select the period and data to download	Accepted
	3	Replace “Symptoms” (from Portuguese *Sintomas*) with “Signs and Symptoms” (from Portuguese *Sinais e Sintomas*)	Accepted
	4	In “Signs and Symptoms,” include a postmastectomy chest wall image	Not accepted
	5	In “Care” (from Portuguese *Cuidados*), replace the checkboxes with radio buttons	Accepted
	6	In “Care,” include information about the water intake goal	Accepted
	7	In “Notes” (from Portuguese *Anotações*), include the possibility of adding photos next to the user’s comments	Accepted
**Schedule**			
	1	Add a screen that highlights the 2 features of this topic (“Events” from Portuguese *Eventos* and “Medicines” from Portuguese *Medicamentos*)	Accepted
	2	In “Events,” allow the exclusion of an event previously included (add an “X” next to the notification symbol)	Accepted
	3	In “Medicines,” allow the exclusion of a drug previously included (add an “X” next to the notification symbol)	Accepted
	4	In “Medicines,” add mechanisms that facilitate their insertion	Accepted
	5	In “Medicines,” add a list of possible drugs to be added	Not accepted
**Settings**			
	1	Add a screen that highlights the 3 features of this topic (“Registration” from Portuguese *Cadastro*, “Tutorial,” and “About the App”)	Accepted
	2	In “About the app,” add “Contact Info” (from Portuguese *Contato*), “Terms of Use” (from Portuguese *Termos de Uso*), and “Privacy Policy” (from Portuguese *Política de Privacidade*)	Accepted

## Discussion

### Main Results

The design of the mobile app “AMOR Mama” was based on an educational manual developed and validated in a previous study named as “Orientations Manual: breast radiation therapy” [11]. The features and functionality of the app were then defined and associated with the textual content of the validated manual. The participants’ judgments were generally consistent (Table 1). Only 1 item of the evaluation tool, which was related to the potentiality of the app to be used by users of different educational levels, obtained a concordance index <80%, considering that 1 expert chose the option “not sure” and another chose the option “in parts” for that item. One of the experts believed that the guidelines presented in the text are not accessible for people with different levels of education. One way to reduce the difficulties in reading and facilitate communication is by using images that are associated with the textual content. Thus, to create a visual representation and illustrate the app, figures and photos obtained in the real treatment environment of patients undergoing radiation therapy were included.

Remote access to health information through mobile technologies contributes to the solution of the patient’s needs without restriction of time and space, thereby promoting the overcoming of barriers and both, the extension of assistance, and the reach of health benefits [18]. In this way, caregivers and family members who are often unable to be present in the treatment environment of patients can help those who have a low level of education, especially in relation to reading and interpreting the information offered by an app that can be accessed freely in any place at any time of the day. Furthermore, a suggestion that emerged during a discussion in one of the focus groups and which was accepted to be implemented in the app was to add explanatory videos related to the stages of radiation therapy, with the health team as actors in a real environment of treatment. This suggestion is very pertinent and useful to complement the textual content and facilitate the understanding for users, especially for those who have a low level of education. In the following study of coding and evaluation of the software, the stages of preparing the scripts, training the team, and recording and editing the explanatory videos to be inserted in the app will also be carried out. Therefore, the content offered by the app must be intelligible, informative, succinct, objective, and delivered to the user in an appropriate manner, that is, through correct texts in relation to spelling and concordance and associated with representative images, which assist in the interpretation of the information provided. The aesthetics must be pleasant, reducing distractions and favoring the interaction between the user and the software [13]. The content must also be constructed consistently. The formatting of the text and its font style, size, and color must be the same throughout all the screens developed, thereby maintaining a standard. In any software design model, consistency is an attribute that must be considered. The graphic design must consist of a pattern of style, aspects, and colors, which provides a consistent interface in relation to the interaction, navigation, and display of content and functionalities [13]. Therefore, a graphic design with well-planned mechanisms provides spontaneous and intuitive interaction with the user. In this study, the interface of the app was designed to be simple, easy to understand, visibly pleasant and attractive, as proposed by a study that focused on the development of an app for teaching the International Classification for Nursing Practice. The development of this app also took into account the importance of attractiveness and objectivity so that some aspects such as the choice of textual content and its font and size as well as the colors of the design provided visual and dynamic comfort in the delivery of information [19]. However, in addition to consistency, the app must have an identity. The aesthetics of the interface must provide affinity with the user to whom the software will be directed [13]. In the elaboration of the app prototype proposed in this study, female aspects were taken into consideration, such as the choice of the predominant color and the images related to the female universe (clothing being illustrated by images of dress and bra and skin care being illustrated by photographs of a woman and female breasts) in addition to the communication being directed to the female gender. Regardless of the information or functionality provided by the app, the screen layout is responsible for shaping the user’s perception of the software, creating a medium of communication between them. In this context, a software user expects to find content and functions relevant to their needs in addition to intuitive and predictable navigation. Without adequate graphic design, an app for mobile devices may be quite functional, but it would not be attractive and, in turn, would not have good usability [13].

Among the features and functionality available in the prototype of the app proposed in this study, we can mention the registration of events in a calendar, with the possibility of generating automatic reminders through notifications with 24 hours in advance. The user’s freedom of choice in relation to enabling or disabling notifications is an important aspect that was already foreseen in the project and was reinforced during the discussion of one of the focus groups. This possibility of adapting to different users is very relevant, considering that an interface must be able to provide flexible interaction since different users may have different preferences for interacting with the app [13]. This customization is also found in the registration where the user can insert a photo and her personal information such as name, weight, date of birth, start date of treatment, and number of sessions planned. Body weight is initially used to calculate the goal of daily water intake, which should be 30 to 35 ml/kg for adults and 25 mL/kg for the elderly, considering the variations according to the symptoms and tolerance presented by each patient [20].

A study also included the possibility of personalization in the development of the proposed app. This app can be useful for recording and monitoring various personal health data such as results of laboratory tests, medications in use, immunization, allergies, body weight, and blood pressure in addition to information related to physical activity, nutrition, and sleep. The modules are customizable and flexible since users can make choices according to their own needs, thereby hiding or showing different information [21]. The suggestion to include links in the app that direct to informative content available on the web was not accepted. The researchers believe that the information already validated by specialists and available on the app is sufficient for the adequate guidance of users. An exaggerated amount of information can be harmful, as suggested by a study that evaluated the effects of an app on the anxiety levels of patients undergoing breast cancer surgery [22].

**Figure 2:**
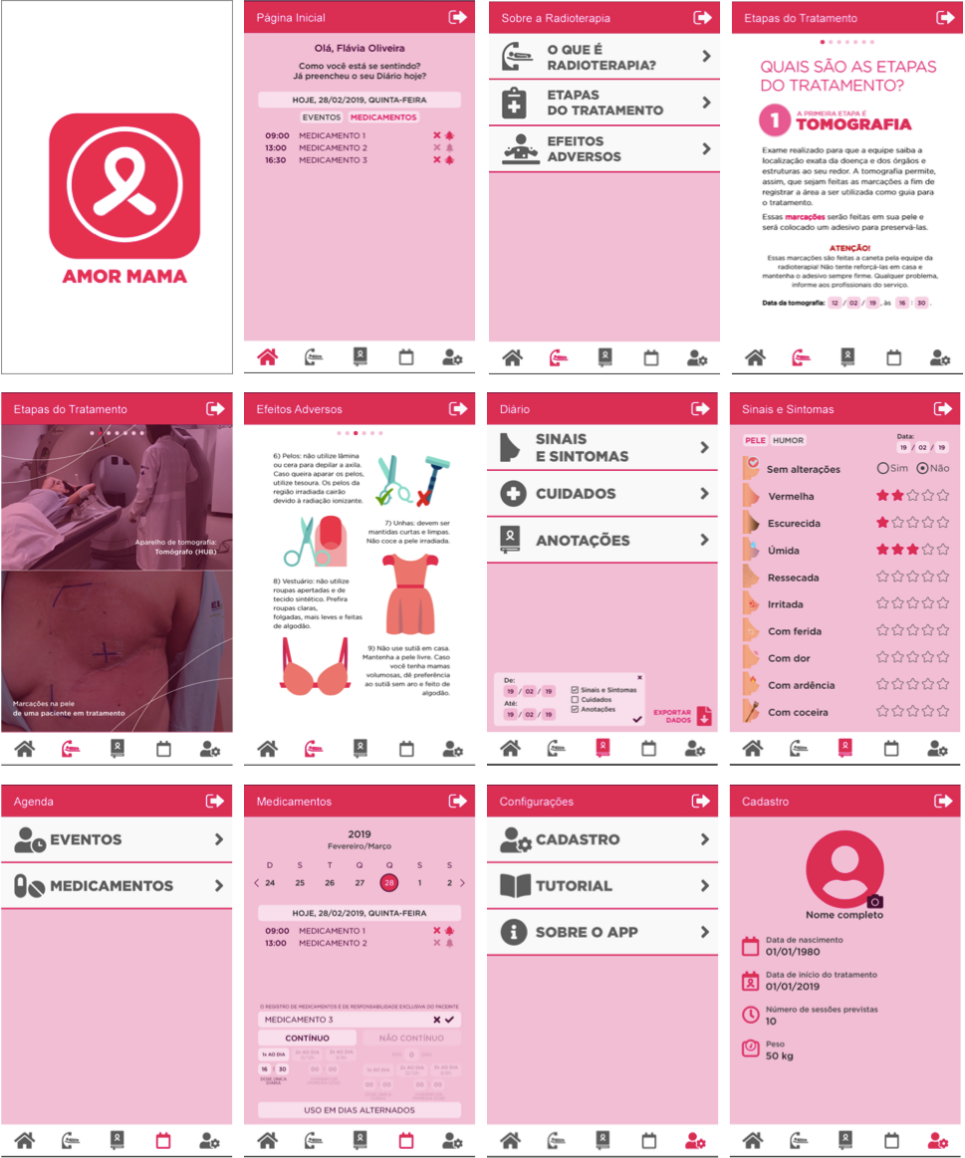
Screenshots of the AMOR Mama app.

The authors of this study also have no control over external content inserted in the app through links and are not responsible for its availability and the updates. In addition, the direction and access to the content available on the web depends on the internet connection, which hurts the initial proposal of offline browsing for the app proposed in this study. Therefore, after making the changes suggested by the participants, the final product of the elaboration and improvement of the app prototype resulted in a model ready to be coded as software, with essential content for patients with breast cancer undergoing radiation therapy and with an interface that favors communication, interactivity, and its use, as shown in Figure 2.

### Limitations

The limitation in this study was related to the difficulty in conducting only 1 focus group, including all participants at once in the design assessment of the mobile app, given the specificities and individual demands of each professional. However, this limitation was overcome by the fact that the focus group was used as a complementary technique to the quantitative assessment, which occurred individually and allowed to obtain objective measures about the evaluation. Another limitation is related to the number of participants included. Despite the recommended minimum of 8 participants, it is believed that a larger sample could have improved the design assessment of the mobile app.

### Conclusions

The mobile app prototype entitled “AMOR Mama,” after having been improved based on the participants’ suggestions, was considered adequate. This indicates its contribution as a technology for health education aimed at the dissemination of guidelines and monitoring of women with breast cancer undergoing radiation therapy. This app will be developed through software codification, and studies with the target population must be carried out to assess the performance and quality of the app during its use.
